# Diagnostic efficacy of serum microRNAs in predicting pathology of retroperitoneal lymph node dissection in patients with testicular germ cell tumors: a systematic review and meta-analysis

**DOI:** 10.1007/s00345-025-05571-y

**Published:** 2025-03-27

**Authors:** Mehdi Kardoust Parizi, Nirmish Singla, Siamak Daneshmand, Axel Heidenreich, Aditya Bagrodia, Vitaly Margulis, Akihiro Matsukawa, Ichiro Tsuboi, Shahrokh F. Shariat

**Affiliations:** 1 Department of Urology, Comprehensive Cancer Center, Vienna General Hospital, Medical University of Vienna, Währinger Gürtel 18-20, 1090 Vienna, Austria; 2 Department of Urology, Shariati Hospital, Tehran University of Medical Sciences, Tehran, Iran; 3 James Buchanan Brady Urological Institute, Johns Hopkins University, Baltimore, MD USA; 4 Department of Urology, University of Southern California Norris Comprehensive Cancer Center, Los Angeles, CA USA; 5 Department of Urology, Uro-Oncology, Robot-Assisted and Specialized Urologic Surgery, University Hospital Cologne, Cologne, Germany; 6 Department of Urology, University of California San Diego, San Diego, CA USA; 7 Department of Urology, University of Texas Southwestern Medical Center, Dallas, TX USA; 8 Department of Urology, Jikei University School of Medicine, Tokyo, Japan; 9 Department of Urology, Faculty of Medicine, Shimane University, Shimane, Japan; 10 Departments of Urology, Weill Cornell Medical College, New York, NY USA; 11 Karl Landsteiner Institute of Urology and Andrology, Vienna, Austria; 12 Department of Urology, Second Faculty of Medicine, Charles University, Prague, Czech Republic; 13 Division of Urology, Department of Special Surgery, The University of Jordan, Amman, Jordan; 14 Research Center for Evidence Medicine, Urology Department, Tabriz University of Medical Sciences, Tabriz, Iran

**Keywords:** MicroRNA, RPLND, MicroRNA-371a-3p, Testicular cancer, Germ cell tumor

## Abstract

**Purpose:**

To evaluate the diagnostic efficacy of serum microRNAs in predicting pathologic findings of retroperitoneal lymph node dissection (RPLND) in patients with testicular germ cell tumors (TGCT).

**Methods:**

PUBMED, SCOPUS, and Cochrane Library were searched in August 2024 to identify eligible studies according to the Preferred Reporting Items for Systematic Reviews and Meta-Analyses (PRISMA 2020) guidelines.

**Results:**

Nine studies comprising 603 patients were selected in this review. The pooled sensitivity, specificity, and diagnostic odds ratio (DOR) of microRNA-371a-3p for predicting viable tumor other than pure teratoma in RPLND specimen were 0.76 (95% CI 0.49–0.90), 0.97 (95% CI 0.81–0.99) and 31.75 (95% CI 9.24–109.10), respectively. The pooled sensitivity for primary and post-chemotherapy RPLND (PC-RPLND), were 0.77 (95% CI 0.47–0.93) and 0.73 (95% CI 0.28–0.95), respectively. The pooled specificity for primary and PC-RPLND were 0.92 (95% CI 0.72–0.98) and 0.99 (95% CI 0.62–1.00), respectively. The pooled DOR for primary and PC-RPLND were 13.86 (95% CI 2.97–64.79) and 64.11 (95% CI 13.09–313.98), respectively. The major limitation is the lack of standardization of miR371 testing.

**Conclusion:**

miR-371a-3p is a relatively sensitive and highly specific marker for predicting viable tumors in RPLND pathologic findings. The DOR was particularly significant for patients who underwent PC-RPLND. While serum microRNAs may be useful in distinguishing viable germ cell tumors from necrosis, fibrosis, and teratomas, their ability to differentiate teratomas from necrosis is limited. Well-designed prospective studies are essential to enhance our understanding of the predictive performance of microRNAs.

**Supplementary Information:**

The online version contains supplementary material available at 10.1007/s00345-025-05571-y.

## Introduction

High survival rates for testicular cancer highlight the effectiveness of current treatments. However, systemic therapies can lead to significant long-term side effects, such as cardiovascular disease, metabolic syndromes, ototoxicity, infertility and secondary malignancies contributing to increased mortality among survivors [1, 2].

Retroperitoneal lymph node dissection (RPLND), whether used in the primary or in post chemotherapy setting, plays a crucial role in the treatment of testicular germ cell tumor (TGCT). Accurate prediction of the presence or absence of viable tumors in retroperitoneal lymph nodes is crucial, particularly since up to 30% of patients with chemotherapy-naïve clinical stage IIA and low-volume stage IIB and up to 50% of post chemotherapy RPLND patients may have nonmalignant disease based on RPLND final histopathologic findings [3–5].

Identifying patients who might benefit from primary RPLND can help minimize treatment-associated toxicity for those with early metastatic disease to regional lymph nodes [1, 5]. The decision-making process regarding the indication for RPLND relies on serum tumor markers (alpha-fetoprotein, beta-human chorionic gonadotropin, and lactate dehydrogenase), as well as imaging studies. However, the inherent limitations of these diagnostic tools may increase the risk of both undertreatment as well as overtreatment for patients. The effectiveness of these markers is constrained by their low sensitivity and specificity for tumor detection. This limitation is influenced by their varied expression across different subtypes and stages of germ cell tumors [6].

Incorporation of molecular markers, could enhance the ability to differentiate between malignant and benign conditions, thereby guiding treatment decisions more effectively. This would not only spare patients from unnecessary interventions but also potentially reduce the risk of long-term complications associated with aggressive therapies.

In early clinical studies, serum microRNAs (in particular miR-371a-3p) demonstrated substantial promise as a biomarker and has been shown to correlate with the extent of disease, response to therapy, relapse, and the presence of residual malignant germ cell tumor elements [7–9]. Therefore, we aimed to investigate the diagnostic values of serum microRNAs in predicting pathologic findings of RPLND in patients with TGCT by systematically reviewing the current literature and meta-analyzing available results.

## Evidence acquisition

### Search strategy

This systematic review and meta-analysis were conducted according to the Preferred Reporting Items for Systematic Reviews and Meta-Analyses (PRISMA) statement [10]. The protocol of this study was a priori registered in PROSPERO, and the protocol is available online (CRD42024597334). We performed a systematic literature search through PUBMED, SCOPUS, and Cochrane Library in August 2024 to identify the eligible studies investigating the serum microRNAs in predicting pathology of RPLND in patients with TGCT. All full text papers were assessed by two reviewers and excluded with reasons when inappropriate after initial screening based on study title and abstract. Disagreements were resolved by consensus with the co-authors. The string terms used in our search strategy were (microRNA OR miRNA OR miR-371a-3p OR microRNAs OR MicroRNA-371a-3p OR miRNAs) AND (testicular germ cell tumor OR testicular cancer OR testis tumor OR testis cancer). The primary endpoint was to assess pooled diagnostic values of miR-371a-3p in predicting viable tumor in RPLND specimens of patients with TGCT. The secondary endpoints were to assess the diagnostic values of other serum miR-371a-3p in the prediction of RPLND pathologic findings.

### Inclusion and exclusion criteria

The population, intervention, control, and outcomes (PICO) in this study were decided by the coauthors as follows: TGCT patients who were undergoing primary or postchemotherapy RPLND and with detected preoperative serum microRNAs compared with negative serum microRNAs in terms of RPLND pathologic finding. Studies were eligible if these reported data on the following: true positive (TP), true negative (TN), false positive (FP), false negative (FN), sensitivity, specificity, accuracy, positive predictive value (PPV), or negative predictive value (NPV). We excluded abstract, replies, editorial comments, review articles, and articles published in other languages than English.

### Data extraction

Two investigators independently extracted the following information from the included articles: study name, publication year, study design, recruitment period, number of patients, primary tumor characteristics, assessed miRNA, clinical indication for RPLND, time of sample collection, miRNA threshold, age, RPLND pathologic report, sensitivity, specificity, PPV, NPV, and test accuracy (AUC; area under the receiver operating characteristic curve).

### Risk of bias assessment

We assessed the risk of bias of included studies according to the revised Quality Assessment of Diagnostic Accuracy Studies tool (QUADAS-2) [11]. MicroRNA-371a-3p and pathologic assessment were defined as index test and standard reference, respectively. Each bias domain and overall risk of bias were judged as “low”, “high”, or “unclear” risk of bias. Disagreements were resolved by consensus or consultation with other authors.

### Statistical analyses

A random effect model was used to estimate pooled sensitivity, specificity, positive likelihood ratio and negative likelihood ratio as well as diagnostic odds ratio for miR-371a-3p. We also performed subgroup analyses for the diagnostic accuracy of miR-371a-3p in predicting RPLND pathologic finding in primary and postchemotherapy RPLND. We created hierarchical summary receiver operating curve (SROC) to examine the differential diagnostic accuracy. We created forest plots with 95% confidence interval (CI) for sensitivity and specificity for each study. Significant heterogeneity was indicated by p < 0.05 in the Cochrane’s Q tests and a ratio of > 50% in I2 statistics. We performed statistical analyses using R version 4.0.3 (2020; R Foundation for Statistical Computing, Vienna, Austria). The statistical significance level was set at p < 0.05.

## Evidence synthesis

### Study selection and characteristics

**Fig. 1:**
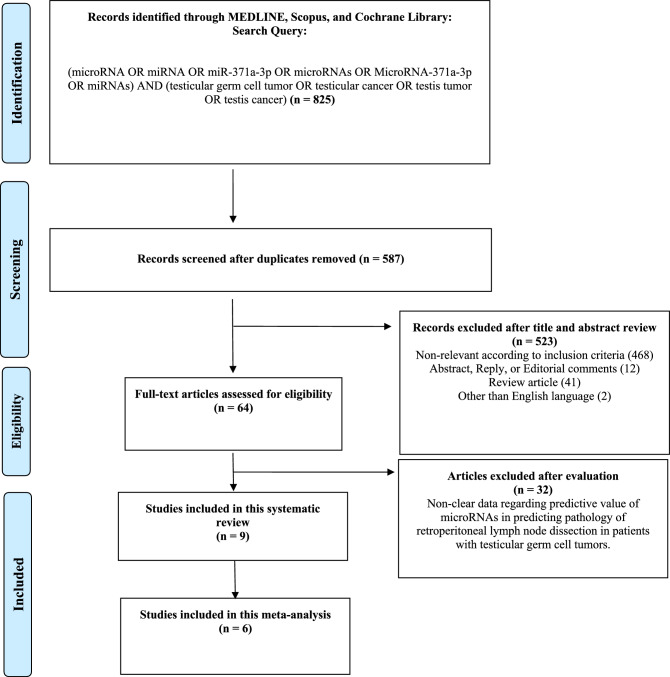
PRISMA flow chart for article selection process to analyze the diagnostic estimates of microRNAs in predicting pathology of retroperitoneal lymph node dissection in patients with testicular germ cell tumors

The literature search identified 825 unique references. Among them, 238 records were removed due to duplication and 523 articles were excluded due to unrelated outcomes during the screening process (Fig. 1). Of the 64 full-text articles assessed for eligibility, 32 were excluded based on the selection criteria. Nine studies were included in the qualitative synthesis [7–9, 12–17]. Eight studies [8, 9, 12–17] were designed prospectively and one [7] was retrospective. The ability of miR-375 to detect teratoma lesions was evaluated in three studies, reporting an AUC ranging from 0.506 to 0.93, sensitivity ranging from 52 to 90%, and specificity ranging from 32 to 71% [9, 15, 16]. Six studies, comprising 400 patients, were included in the quantitative synthesis. All the studies included in the quantitative synthesis reported on the diagnostic value of miR-371a-3p for the prediction of RPLND pathologic finding [7, 12–14, 16, 17]. Tables 1 and 2 summarize the characteristics of the studies and patients’ clinical data.

**Table 1:** Characteristics of included studies reporting the diagnostic estimates of microRNAs in predicting pathology of retroperitoneal lymph node dissection in patients with testicular germ cell tumors

Study	Year	Study design	Recruitment period	Pts	Primary tumor (%)	Assessed microRNA	Clinical indication for RPLND (%)	Time of serum samples collection before surgery	MicroRNA threshold
Leão^7^	2018	Retrospective	2009–2016	82	NSGCT (100)	miRNA-371a-3p, miRNA-373-3p, miRNA-367-3p	Post chemotherapy (100)	NA	miRNA-371a-3p: 2
Lafin^8^	2020	Prospective	2016–2019	24	NSGCT (75), Seminoma (17), Benign (8)	miRNA-371a-3p, miRNA-367-3p, miRNA-372-3p, miRNA-373-3p, miRNA-375	Primary (100)	Within 10 days of RPLND	NA
Lafin^16^	2021	Prospective	2016–2019	40	NSGCT (100)	miRNA-375-3p, miRNA-375-5p	Post chemotherapy (100)	Immediately prior to RPLND	miR-375-3p: 0.6, miR-375-5p: 1.16
Nappi^9^	2021	Prospective	NA	100	NSGCT (64), Seminoma (34), Burned out (1), Leydig tumor (1)	miRNA-371a-3p, miRNA-375	Post chemotherapy (58), Primary (42)	NA	8
Konneh^17^	2023	Prospective	2016–2020	15	Seminoma (100)	miR-371a-3p	Post chemotherapy (33), Primary (67)	Within 28 days of RPLND	28.62
Moore^15^	2023	Prospective	NA	22	NSGCT (100)	miRNA-375, miRNA-200a-3p, miRNA-200a-5p, miRNA-200b-3p	Post chemotherapy (100)^a^	Within 3 months prior to consolidation surgery	NA
Dieckmann^14^	2024	Prospective	2018–2023	180	NSGCT (92), Seminoma (8)	miRNA-371a-3p	Post chemotherapy (100)^b^	NA	5.6
Seelemeyer^13^	2024	Prospective	2022–2023	26	NSGCT (35), Seminoma (65)	miRNA-371a-3p	Primary (100)	On the day before RPLND	10
Thor^12^	2024	Prospective	2017–2022	114	NSGCT (75), Seminoma (25)	miRNA-371a-3p	Post chemotherapy (63), Primary (37)	Up to 1 week prior to RPLND	0.45

*NA* not available, *NSGCT* non-seminomatous germ cell tumor, *miRNA* microRNA, *RPLND* retroperitoneal lymph node dissection

^a^Postchemotherapy consolidation surgery (mostly post-chemotherapy retroperitoneal lymph node dissections)

^b^Postchemotherapy residual masses resection

**Table 2:** Diagnostic performance of microRNAs in predicting pathology of retroperitoneal lymph node dissection in patients with testicular germ cell tumors

Study	Age, year	RPLND pathology (%)	Sensitivity (%)			Specificity (%)			PPV (%)			NPV (%)			AUC		
			VT	T	BNF	VT	T	BNF	VT	T	BNF	VT	T	BNF	VT	T	BNF
Leão^7^	Median: 26	BNF (43.9), T (41.5), VT (14.6)	miR-371a-3p: 100^a^	miR-371a-3p: 38	miR-371a-3p: 50	miR-371a-3p: 54^a^	miR-371a-3p: 38	miR-371a-3p: 42	miR-371a-3p: 28	miR-371a-3p: 24	miR-371a-3p: 48	miR-371a-3p: 100	miR-371a-3p: 55	miR-371a-3p: 44	miR-371a-3p/miR-373-3p: 0.885; 95% CI 0.79–0.98^b^	NA	NA
Lafin^8^	Median: 27	BNF (41.7), T (12.5), VT (45.8)	miR-371a-3p: 100	–	miR-371a-3p: 25	miR-371a-3p: 92	miR-371a-3p: 43	miR-371a-3p: 45	miR-371a-3p: 92	–	miR-371a-3p: 8	miR-371a-3p: 92	miR-371a-3p: 75	miR-371a-3p: 75	miR-371a-3p: 0.965^c^	NA	NA
Lafin^16^	Median: 25.5	BNF (47.5), T (47.5), VT (5)	NA	miR-375-3p: 86, miR-375-5p: 55	NA	NA	miR-375-3p: 32, miR-375-5p: 67	NA	NA	miR-375-3p: 58	NA	NA	miR-375-3p: 67	NA	NA	miR-375-3p: 0.506; 95% CI 0.32–0.69. miR-375-5p: 0.556; 95% CI 0.30–0.81	NA
Nappi^9^	NA	BNF (38), T (41), VT (21)	NA	miR375: Discovery cohort: 90, Validation cohort: 52	NA	NA	miR375: Discovery cohort: 81, Validation cohort: 70	NA	NA	miR375: Discovery cohort: 69, Validation cohort: 68	NA	NA	miR375: Discovery cohort: 94, Validation cohort: 54	NA	NA	Discovery cohort: miR375: 0.93; 95% CI: 0.87–0.99. miR371: 0.59; 95% CI: 0.44–0.73. miR371-miR375: 0.95; 95% CI: 0.90–0.99. Validation cohort: miR375: 0.55; 95% CI: 0.36–0.74. miR371: 0.74; 95% CI: 0.58–0.91. miR371-miR375: 0.77; 95% CI: 0.62–0.93	NA
Konneh^17^	Median: 33	BNF (40), VT (60)	miR-371a-3p: All patients: 78, Primary RPLND: 78	NA	NA	miR-371a-3p: All patients: 67, Primary RPLND: 100, Post chemotherapy RPLND: 60	NA	NA	miR-371a-3p: Primary RPLND: 100	NA	NA	miR-371a-3p: Primary RPLND: 33, Post chemotherapy RPLND: 100	NA	NA	miR-371a-3p: All patients: AUC 0.704; 95% CI: 0.43–0.98	NA	NA
Moore^15^	Mean: T: 30.8, VT and BFN: 26.5	BNF and VT (50), T (50)	NA	NA	NA	NA	NA	NA	NA	NA	NA	NA	NA	NA	NA	miRNA-375: 0.619, miRNA-200a-5p: 512, miRNA-200a-3p: 0.719, miRNA-200b-3p: 0.537	NA
Dieckmann^14^	Median: 31	BNF (33), T (42), VT (25)	miRNA-371a-3p: 68.9	–	miRNA-371a-3p: 2	miRNA-371a-3p: 99.3	miRNA-371a-3p: 69	miRNA-371a-3p: 74	miRNA-371a-3p: 97	–	miRNA-371a-3p: 3	miRNA-371a-3p: 90	miRNA-371a-3p: 49	miRNA-371a-3p: 60	miRNA-371a-3p: 0.813; 95% CI 0.721–0.905	NA	NA
Seelemeyer^13^	Median: 38.7	BNF (8), VT (92)	miRNA-371a-3p: 90.9	–	–	miRNA-371a-3p: 50	miRNA-371a-3p: 20	miRNA-371a-3p: 9	miRNA-371a-3p: 100	–	–	miRNA-371a-3p: 75	miRNA-371a-3p: 83	miRNA-371a-3p: 33	NA	NA	NA
Thor^12^	Median: 29	BNF (28), T (39), VT (33)	miRNA-371a-3p: 74^d^, 34^e^, 9^f^	miRNA-371a-3p: 14^e^	–	miRNA-371a-3p: 100^d^, 88^e^, 100^f^	miRNA-371a-3p: 73^e^, 97^f^	miRNA-371a-3p: 26^d^, 73^e^, 98^f^	miRNA-371a-3p: 100^d^, 67^e^, 100^f^	miRNA-371a-3p: 25^e^	–	miRNA-371a-3p: 21^d^, 62^e^, 64^f^	miRNA-371a-3p: 57^e^, 48^f^	miRNA-371a-3p: 86^d^, 78^e^, 62^f^	NA	NA	NA

*NA* not available, *RPLND* retroperitoneal lymph node dissection, *PPV* positive predictive value, *NPV* negative predictive value, *VT* viable tumor, *T* teratoma, *BFN* benign, fibrosis, necrosis, *AUC* area under the ROC curve, *CI* confidence interval

^a^miR-371a-3p/miR-373-3p signature identifies viable tumor with sensitivity of 100% and specificity of 58% (*p* = 0.02)

^b^miR-371a-3p/miR-373- 3p/miR-367: AUC 0.880; 95% CI 0.79–0.99. miR-371a-3p: AUC 0.874; 95% CI 0.77–0.97. miR-371a-3p/miR-367: AUC 0.873; 95% CI 0.78–0.98. miR-373- 3p: AUC 0.738; 95% CI 0.59–0.88. miR-367: AUC 0.707; 95% CI 0.54–0.87

^c^miR-367-3p: AUC 0.874, miR-372-3p: AUC 0.874, miR-373- 3p: AUC 0.720

^d^Seminoma primary retroperitoneal lymph node dissection group

^e^Nonseminoma primary retroperitoneal lymph node dissection group

^f^Postchemotherapy retroperitoneal lymph node dissection group

### Meta-analysis of serum miR-371a-3p predicting RPLND pathologic finding

#### Viable tumor excluding pure teratoma

**Fig. 2:**
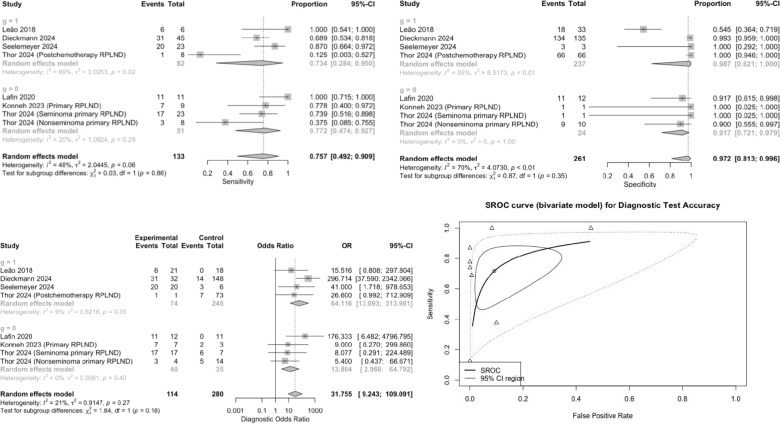
Forest plot for pooled Sensitivity, pooled Specificity, pooled diagnostic odds ratio, and SROC curve of microRNA-371a-3p in predicting viable tumor (excluding pure teratoma) in RPLND specimen of patients with testicular germ cell tumors. g = 0; primary RPLND. g = 1; postchemotherapy RPLND. *CI* confidence interval, *SROC* summary receiver operating characteristics, *OR* odds ratio, *RPLND* retroperitoneal lymph node dissection

Six studies provided data on the diagnostic values of serum miR-371a-3p for predicting of viable tumor excluding teratoma in the RPLND specimen [7, 8, 12–14, 17]. The pooled sensitivity and specificity were 0.76 (95% CI 0.49–0.90) and 0.97 (95% CI 0.81–0.99), respectively (Fig. 2). The pooled DOR was 31.75 (95% CI 9.24–109.10). The Cochrane’s Q tests and I2 tests revealed significant heterogeneity among studies for pooled specificity. The test reached an AUC of 0.876 for the detection of viable tumor excluding pure teratoma. In the subgroup analysis of patients who underwent primary RPLND, the forest plots revealed that the pooled sensitivity and specificity were 0.77 (95% CI 0.47–0.93) and 0.92 (95% CI 0.72–0.98), respectively. The Cochrane’s Q tests and I2 tests revealed no significant heterogeneity among studies. The pooled DOR was 13.86 (95% CI 2.97–64.79). In the subgroup analysis of patients who underwent post-chemotherapy RPLND (PC-RPLND), the forest plots revealed that the pooled sensitivity and specificity were 0.73 (95% CI 0.28–0.95) and 0.99 (95% CI 0.62–1.00), respectively. The pooled DOR was 64.11 (95% CI 13.09–313.98). The Cochrane’s Q tests and I2 tests revealed significant heterogeneity among studies for pooled sensitivity and pooled specificity.

#### Viable tumor including pure teratoma

**Fig. 3:**
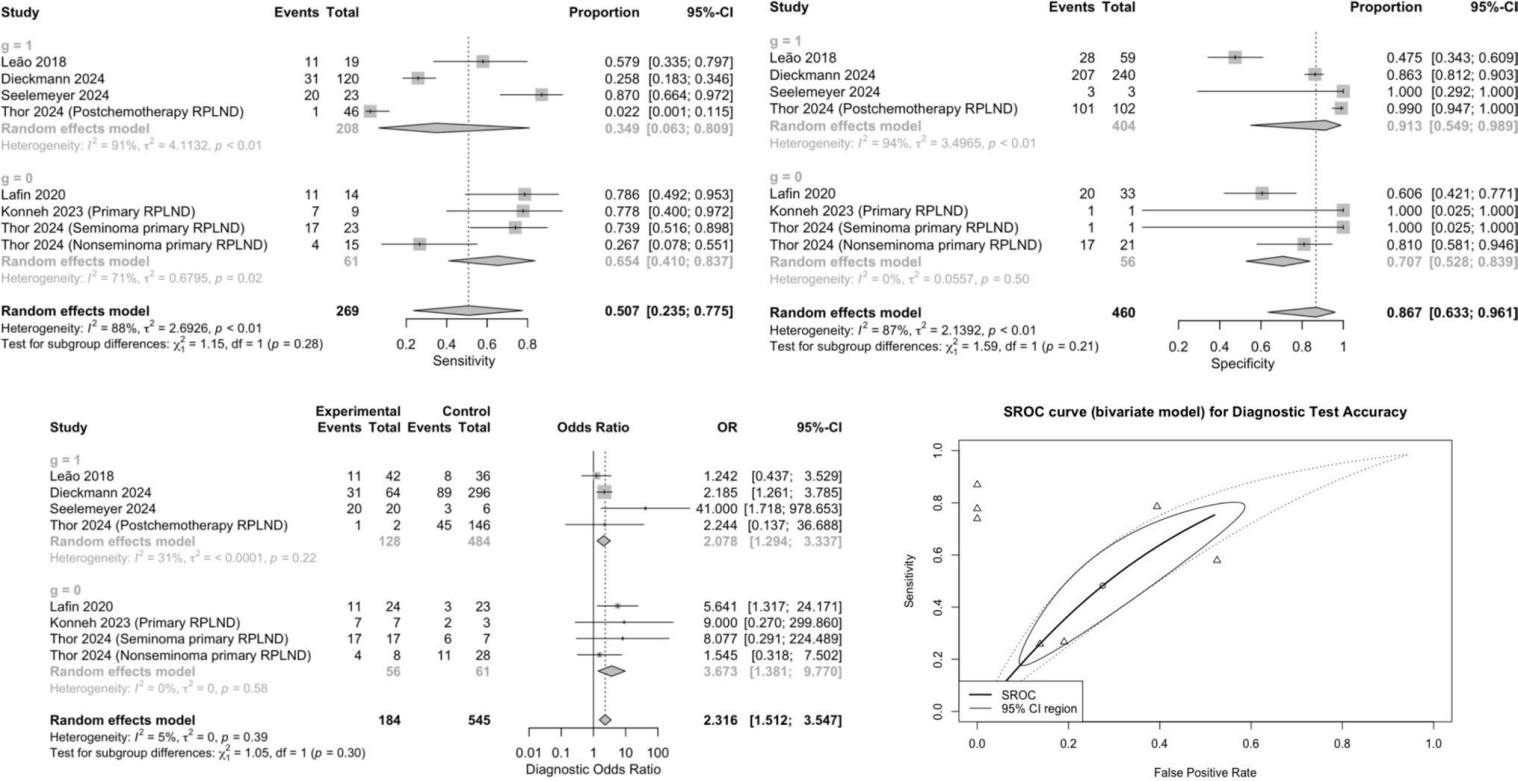
Forest plot for pooled Sensitivity, pooled Specificity, pooled diagnostic odds ratio, and SROC curve of microRNA-371a-3p in predicting viable tumor (including pure teratoma) in RPLND specimen of patients with testicular germ cell tumors. g = 0; primary RPLND. g = 1; postchemotherapy RPLND. *CI* confidence interval, *SROC* summary receiver operating characteristics, *OR* odds ratio, *RPLND* retroperitoneal lymph node dissection

Six studies provided data on the diagnostic values of serum miR-371a-3p for predicting viable tumor including teratoma in RPLND specimen [7, 8, 12–14, 17]. The pooled sensitivity and specificity were 0.51 (95% CI 0.23–0.77) and 0.87 (95% CI 0.63– 0.96), respectively (Fig. 3). The pooled DOR was 2.32 (95% CI 1.51–3.55). The Cochrane’s Q tests and I2 tests revealed significant heterogeneity among studies for pooled sensitivity and pooled specificity. The test reached an AUC of 0.652 for the detection of viable tumor including pure teratoma. In the subgroup analysis of patients who underwent primary RPLND, the forest plots revealed that the pooled sensitivity and specificity were 0.65 (95% CI 0.41–0.84) and 0.71 (95% CI 0.53–0.84), respectively. The pooled DOR was 3.67 (95% CI 1.38–9.77). The Cochrane’s Q tests and I2 tests revealed significant heterogeneity among studies for pooled sensitivity. In the subgroup analysis of patients who underwent PC-RPLND, the forest plots revealed that the pooled sensitivity and specificity were 0.35 (95% CI 0.06–0.81) and 0.91 (95% CI 0.55–0.99), respectively. The pooled DOR was 2.08 (95% CI 1.29–3.34). The Cochrane’s Q tests and I2 tests revealed significant heterogeneity among studies. Supplementary Fig. 1 showed funnel plots of DOR for microRNA-371a-3p in predicting RPLND pathologic findings.

### Risk of bias assessment

The summary of the risk of bias and applicability concerns is presented in supplementary Table 1 with low to intermediate risk of bias in nine studies. Overall quality of the included studies was deemed satisfactory.

## Discussion

We conducted the first meta-analysis of the diagnostic estimates of microRNA-371a-3p for predicting pathologic findings of RPLND in patients with TGCT. Our results demonstrate that microRNA-371a-3p has good performance characteristics for the detection of germ cell tumor (GCT).

The capacity of microRNA-371a-3p to accurate differentiation of low-volume viable GCT from benign processes in patients undergoing primary RPLND is helpful to accurately personalize the decision to forego or pursue primary treatment (RPLND, chemotherapy, or radiotherapy) in patients arguably best suited for surveillance would mitigate the morbidity associated with overtreatment. Teratoma is negative for miR371, however, it needs active treatment. Therefore, positive miR371 might be helpful in marker negative stage IIA/B seminoma and in nonseminomas which do not have teratoma in the primary orchiectomy specimens [1, 18]. However, these results might be limited due to the number of patients. Furthermore, we found that microRNA-371a-3p could not accurately predict pure teratoma at RPLND adding another limitation for this biomarker for clinical decision-making particularly in the post-chemotherapy setting of residual retroperitoneal mass. Further prospective research is needed to validate the potential role of microRNA-371a-3p as an alternative to traditional biomarkers in patients with early-stage TGCT. One such study, the MAGESTIC trial, is currently in progress in the United States. This trial, (*MAGESTIC-MiRNA in detecting Active Germ cell tumors in Early Suspected and metastaTIC disease)*, seeks to explore miR-371’s relevance in routine clinical practice (NCT06060873). This investigator-initiated biomarker-guided surgical trial aims to validate the role of miR-371 in guiding treatment decisions for early-stage, conventional tumor marker–negative TGCTs [19]. The SWOG S1823 trial is a clinical study focused on evaluating the effectiveness of active surveillance guided by microRNA 371 (miR-371) levels in patients with early-stage testicular germ cell tumors. The trial aims to determine whether monitoring miR-371 can improve early detection of relapse and reduce the need for traditional imaging and tumor marker tests, potentially leading to more precise and less invasive management of these patients [20].

Our subgroup analysis on the detection of the post chemotherapy viable residual GCT demonstrated similar diagnostic estimates to those in the overall population. Detecting viable residual GCT would be helpful in distinguishing patients with necrosis only, who may be eligible for active surveillance instead of undergoing PC-RPLND, as approximately 40–45% of patients may experience overtreatment with PC-RPLND due to the presence of necrosis [21, 22]. However, this distinction is challenging because necrosis cannot currently be distinguished from teratoma, which is chemoresistant and necessitates surgical management. Our meta-analysis demonstrated this by including viable GCT and teratoma when differentiating them from benign pathology and necrosis, revealing lower diagnostic estimates compared to analyses focused solely on viable GCT. Our findings suggest that although serum miR-375 initially showed to be a promising biomarker for detecting teratomas, its utility is hampered by low positive and negative predictive values, highlighting the pressing need for additional more reliable biomarkers for teratoma [9, 16]. Combining these microRNAs with additional markers, such as messenger RNA and other molecular indicators, could improve the sensitivity and specificity of teratoma detection. Additionally, Advancements in artificial intelligence, especially through radiomics, can greatly improve the analysis of complex biomarker profiles. By combining imaging data with molecular biomarkers, artificial intelligence enhances the accuracy and reliability of diagnostic tools for identifying teratomas. Machine learning algorithms can detect subtle patterns in large datasets, leading to more precise diagnoses and personalized treatment strategies, ultimately improving patient outcomes in teratoma management. Our study is not devoid of limitations. First, the major limitation is the lack of standardization of miR371 testing and the difference in the methods between groups resulting in significant differences of sensitivity and specificity. Studies have used different protocols, such as quantitative reverse transcriptase PCR (RT-qPCR) and droplet digital PCR (ddPCR), which produce varying results due to differences in detection capabilities and normalization methods. RT-qPCR often relies on housekeeping microRNAs for normalization, leading to relative quantification that may differ between studies. In contrast, ddPCR provides absolute quantification and is less affected by inhibitors, but its application for miRNA quantification remains largely underexplored [23, 24]. We identified a high risk of bias in two studies [7, 14] concerning flow and timing, characterized by irregular test sequencing, potential participant exclusions, and inconsistent follow-up processes. These methodological limitations could potentially compromise the study’s internal validity. However, the low number of cases in one study [7] and its correspondingly reduced statistical weight suggest that the potential bias might have minimal impact on the overall meta-analysis results. Therefore, due to the potential biases identified across the studies, the meta-analysis results warrant a careful and critical interpretation, acknowledging the limitations inherent in the current dataset. Also, reporting bias could have led to the nonpublication of negative results. Then, and the lack of standardized threshold for the assessment of serum microRNAs and the heterogeneity of pre-analytical variables such as collection tubes, time to processing the sample, extraction kits and pipelines, have to be mentioned. Furthermore, heterogeneity was observed in both primary tumor characteristics (seminoma versus nonseminoma) and the clinical stage, alongside limited data on RPLND details, thus limiting the value of these results. The lack of clear data regarding the template and the number of resected lymph nodes, as well as insufficient information on the relationship between the extension of RPLND and the results of miRNA assessment may affect the interpretation and applicability of our findings. In addition, the small sample size of the included studies for quantitative synthesis may impact the overall quality of data.

## Conclusion

Our study highlights the potential of serum microRNAs in predicting RPLND pathology, particularly in distinguishing between viable germ cell tumors and benign conditions, including necrosis and teratomas, using microRNA-371a-3p. However, the data available for differentiating teratomas from necrosis is limited. Further well-designed, prospective studies are underway to clarify the predictive performance of microRNAs, which could aid in clinical decision-making for patients with TGCT.

## Supplementary Information

Below is the link to the electronic supplementary material.

Supplementary file1 (PDF 110 KB)

Supplementary file2 (DOCX 15 KB)

## Data Availability

No datasets were generated or analysed during the current study.
