# Supplementary material for: Diagnostic efficacy of serum microRNAs in predicting pathology of retroperitoneal lymph node dissection in patients with testicular germ cell tumors: a systematic review and meta-analysis
Source: World J Urol. 2025 Mar 27;43(1):192. doi: 10.1007/s00345-025-05571-y (PMC11950128; doi:10.1007/s00345-025-05571-y)
Supplement: Supplementary file 1 — Supplementary file1 (PDF 110 KB) [file 345_2025_5571_MOESM1_ESM.pdf]

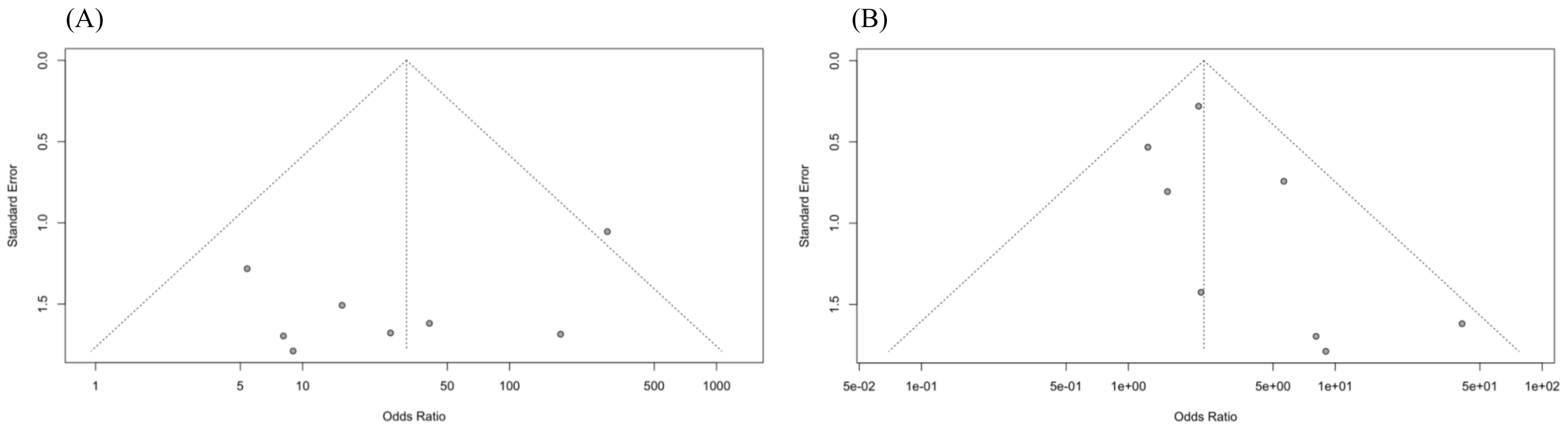

Supplementary Fig. 1. Funnel plots of diagnostic odds ratio of of microRNA-371a-3p in predicting viable tumor (A: excluding pure teratoma, B: including pure teratoma) in retroperitoneal lymph node dissection specimen of patients with testicular germ cell tumors.
