# Supplementary material for: Diagnostic efficacy of serum microRNAs in predicting pathology of retroperitoneal lymph node dissection in patients with testicular germ cell tumors: a systematic review and meta-analysis
Source: World J Urol. 2025 Mar 27;43(1):192. doi: 10.1007/s00345-025-05571-y (PMC11950128; doi:10.1007/s00345-025-05571-y)
Supplement: Supplementary file 2 — Supplementary file2 (DOCX 15 KB) [file 345_2025_5571_MOESM2_ESM.docx]

| Supplementary Table 1. Risk of bias assessment using Quality Assessment of Diagnostic Accuracy Studies-2 (Quadas-2) | | | | | | | |
| --- | --- | --- | --- | --- | --- | --- | --- |
| Study, Year | Risk of bias | | | | Applicability concerns | | |
|  | Patient selection | Index test | Reference standard | Flow and timing | Patient selection | Index test | Reference standard |
| Leão 2018 | High | Low | Unclear | High | Low | Low | Unclear |
| Lafin 2020 | Unclear | High | Low | Unclear | Low | Unclear | Unclear |
| Lafin 2021 | Low | Low | Low | Low | High | Low | Low |
| Nappi 2021 | Low | Low | Low | High | Unclear | Low | Low |
| Konneh 2023 | Low | Low | Low | Unclear | Low | Unclear | Unclear |
| Moore 2023 | Low | High | Low | Unclear | Low | Unclear | Unclear |
| Dieckmann 2024 | Low | Low | Low | High | Low | Low | Low |
| Seelemeyer 2024 | Low | Low | Low | Unclear | Low | Low | Unclear |
| Thor 2024 | Low | Unclear | Low | Low | Low | Low | Unclear |
